# The Impact of Carboplatin Dosing Design Using Adjusted Serum Creatinine on Carboplatin Plus Paclitaxel Therapy for Ovarian Cancer

**DOI:** 10.1002/cam4.70804

**Published:** 2025-03-27

**Authors:** Shu Kato, Kaede Baba, Kanako Mamishin, Mao Uematsu, Mai Shimura, Akira Hirota, Misao Fukuda, Nobuyuki Takahashi, Takehiro Nakao, Hiromichi Nakajima, Chikako Funasaka, Chihiro Kondoh, Kenichi Harano, Yoichi Naito, Nobuaki Matsubara, Ako Hosono, Toshikatsu Kawasaki, Toru Mukohara

**Affiliations:** ^1^ Department of Pharmacy National Cancer Center Hospital East Kashiwa Japan; ^2^ Department of Medical Oncology National Cancer Center Hospital East Kashiwa Japan; ^3^ Department of Experimental Therapeutics National Cancer Center Hospital East Kashiwa Japan; ^4^ Department of General Internal Medicine National Cancer Center Hospital East Kashiwa Japan; ^5^ Department of Pediatric Oncology National Cancer Center Hospital East Kashiwa Japan

**Keywords:** adjuvant chemotherapy, carboplatin, creatinine, ovarian cancer, paclitaxel

## Abstract

**Background:**

Carboplatin (CBDCA) is a mainstay of chemotherapy for ovarian cancer and its dose is determined in proportion to the estimated creatinine clearance (CCr). Serum creatinine (SCr) values necessary to estimate CCr vary by measurement method: adding 0.2 mg/dL to SCr by enzymatic methods commonly used in Japan equates to SCr calculated using the Jaffe method, which is widely adopted outside Japan. Although adjustment by adding 0.2 mg/dL to SCr by enzymatic methods may avoid the potential overdose of CBDCA, its impact on the dose intensity (DI) of chemotherapy is unclear.

**Methods:**

We retrospectively studied patients with ovarian cancer treated with CBDCA + paclitaxel (PTX) (TC) after primary surgery. Patients were classified into Cohort A (dose‐dense [dd‐]TC, SCr‐adjusted, *n* = 18), B (dd‐TC, non‐adjusted, *n* = 8), C (tri‐weekly [tw‐]TC, SCr‐adjusted, *n* = 6), and D (tw‐TC, non‐adjusted, *n* = 15), and DI and DI‐related measures including average relative DI (ARDI, [RDI of CBDCA + RDI of PTX]/2]) known to correlate with patients’ prognoses were compared.

**Results:**

Although the DI of CBDCA did not differ between Cohorts A and B, the DI of PTX and proportion of patients with ARDI ≥ 85% were higher in Cohort A than B (78 vs. 13%, *p* = 0.002) as a result of less frequent treatment modification. There was no difference in these measures between Cohorts C and D.

**Conclusion:**

Adjustment of SCr when calculating the CBDCA dose did not compromise the DI of total CBDCA and may rather contribute to maintaining DI in patients receiving dd‐TC.

## Graphical abstract

This retrospective study aimed to assess the impact of adjusting serum creatinine (SCr) values when determining carboplatin (CBDCA) doses in ovarian cancer chemotherapy. Patients treated with CBDCA + paclitaxel (TC) were categorized based on the regimen they received (dose‐dense TC or tri‐weekly TC) and whether or not their SCr levels were adjusted. Our findings suggest that in patients receiving dose‐dense TC, but not in those receiving tri‐weekly TC, adjusting SCr did not compromise the dose intensity of total CBDCA and may contribute to maintaining dose intensity, highlighting potential benefits in reducing treatment modification and adverse events.

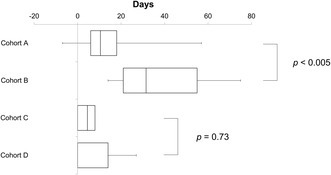

**Abbreviations**
ARDI: average relative dose intensity
AUC: area under the curve
CBDCA: Carboplatin
CCr: creatinine clearance
CTCAE: Common Terminology Criteria for Adverse Events
dd‐TC: dose‐dense carboplatin + paclitaxel
DI: dose intensity
G‐CSF: granulocyte colony stimulating factor
GFR: glomerular filtration rate
IDS: interval debulking surgery
PDS: primary debulking surgery
PS: performance status
RDI: relative dose intensity
SCr: serum creatinine
tw‐TC: tri‐weekly carboplatin + paclitaxel

## 1 Introduction

Ovarian cancer has the highest mortality rate among gynecological malignancies. Many cases of ovarian cancer are advanced at the time of diagnosis due to a lack of subjective symptoms: III and IV account for more than 40% of cases and their prognosis is poor. The number of patients in Japan is increasing, with 13,388 cases reported in 2019 [1].

The initial treatment of Stages II–IV ovarian cancer is mainly delivered as a multimodality treatment combining surgery and chemotherapy [2]. In cases where primary debulking surgery (PDS) is possible, six cycles of postoperative chemotherapy with carboplatin (CBDCA) + paclitaxel (PTX) (TC) with or without bevacizumab, a monoclonal antibody against vascular endothelial growth factor, are generally administered after PDS [3]. In cases where PDS is not feasible, three to six cycles of neoadjuvant chemotherapy with TC with or without bevacizumab followed by interval debulking surgery (IDS) and additional post‐IDS chemotherapy are an option [4]. Patients with recurrent disease that occurs longer than 6 months after the last administration of CBDCA, which is called platinum‐sensitive disease, are generally treated with a CBDCA‐containing regimen [5]. Therefore, CBDCA is considered to be the mainstay of drug treatment for ovarian cancer.

The dose‐limiting toxicity of CBDCA is hematologic toxicity, mainly thrombocytopenia [6]. When combined with paclitaxel, that is, as a TC regimen, neutropenia becomes more prominent as an overlapping toxicity of both drugs. The incidences of grade 3 or greater neutropenia and thrombocytopenia in the Common Terminology Criteria for Adverse Events (CTCAE) version 5.0 by conventional tri‐weekly (tw‐)TC were 88% and 38%, respectively [7]. In a randomized study of dose‐dense (dd‐)TC, in which PTX was administered weekly to increase treatment intensity, compared with tw‐TC, the incidences of grade 3 or greater neutropenia and thrombocytopenia were similarly high in both arms [7]. Moreover, a retrospective study demonstrated that the overall survival was significantly shorter in individuals with a relative dose intensity (RDI) < 85% in post‐PDS chemotherapy than those with an RDI ≥ 85% [8]. Furthermore, average relative dose intensity (ARDI), [(RDI of CBDCA + RDI of PTX)/2], in post‐PDS chemotherapy was reported to correlate with the prognosis of patients with ovarian cancer [8, 9]. Therefore, managing hematologic toxicities from CBDCA‐based combination chemotherapies to maintain dose intensity (DI) is a critical issue in the treatment of ovarian cancer [9].

Because the clearance of CBDCA is proportional to the granulocyte colony stimulating factor (GFR) and the area under the curve (AUC) of CBDCA positively correlates with the incidence of grade 3 or higher thrombocytopenia, the Calvert formula [dose = target AUC × (GFR + 25)] is commonly used to optimize the dose of CBDCA [10, 11]. In clinics, creatinine clearance (CCr) is used as a surrogate of GFR. This is recommended by the US FDA and is widely used in routine clinical practice globally [12]. One of the formulas most widely accepted for predicting CCr is the Cockcroft‐Gault formula: CCr = {[(140 − age) × body weight (kg)]/[72 × serum creatinine (SCr) (mg/dL)]} × 0.85 (if female).

However, values of SCr can differ when using different measurement methods [13]. Enzymatic methods, which are commonly used in Japan, were reported to provide 0.2 mg/dL lower values than when measured by the Jaffe method [13], which is widely adopted in Europe and the US. This means that SCr determined by enzymatic methods may result in a higher CCr and consequently a higher dose of CBDCA, compared with using SCr by the Jaffe method. Indeed, a higher incidence of grade 3/4 thrombocytopenia was reported in clinical trials in which a dose of CBDCA was set based on SCr by enzymatic methods compared with those using the Jaffe method [14]. Based on these findings, adding 0.2 mg/dL to SCr by enzymatic methods is suggested as an adjustment in “Clinical practice guidelines for the management of kidney injury during anticancer drug therapy 2022” issued by the Japanese Society of Nephrology [15]. However, the adjustment has not been fully adopted in clinical practice in Japan and it is left to the physician's discretion, because no reports have directly evaluated the impact of the adjustment on the efficacy and safety of chemotherapy for ovarian cancer.

This study evaluated the impact of the adjustment of SCr by adding 0.2 mg/dL to the DI and safety of chemotherapy for ovarian cancer. We hypothesized that the SCr adjustment may avoid serious toxicities by CBDCA‐containing chemotherapy and thus enable treatment to continue without dose reduction, postponement, or skipping, resulting in a higher DI than in cases without the adjustment. To test the hypothesis, we compared the absolute DIs of CBDCA and PTX, and other DI‐related and safety measures, between patient cohorts with SCr adjustment and without, in patients with ovarian cancer who underwent TC therapy as post‐PDS chemotherapy.

## 2 Materials and Methods

### 2.1 Study Design and Patients

This study retrospectively analyzed data from the medical records of consecutive patients with ovarian cancer who received TC (tw‐TC or dd‐TC) after PDS at the National Cancer Center Hospital East from 1st January 2014 to 31st December 2021. We collected the following information from the medical records: patients' background including age, height, weight, performance status (PS), clinical and pathological stages, and tumor histology; chemotherapy dosing including the presence or absence of the adjustment of SCr in the dose setting of CBDCA, doses of CBDCA and PTX actually administered, dates of the administration, the timing of, and reasons for, dose interruption, dose reduction, and discontinuation; laboratory values including SCr, complete blood cell count, and tumor markers; and other non‐hematologic toxicities. The grades of adverse events were categorized based on CTCAE version 5.0. Patients with CCr estimations other than the Cockcroft‐Gault formula and SCr adjustment other than adding 0.2 mg/dL were excluded. The SCr adjustment was adopted for most patients after 2020 when a consensus meeting of our clinical team agreed to use it.

### 2.2 Patient Cohorts

**FIGURE 1:**
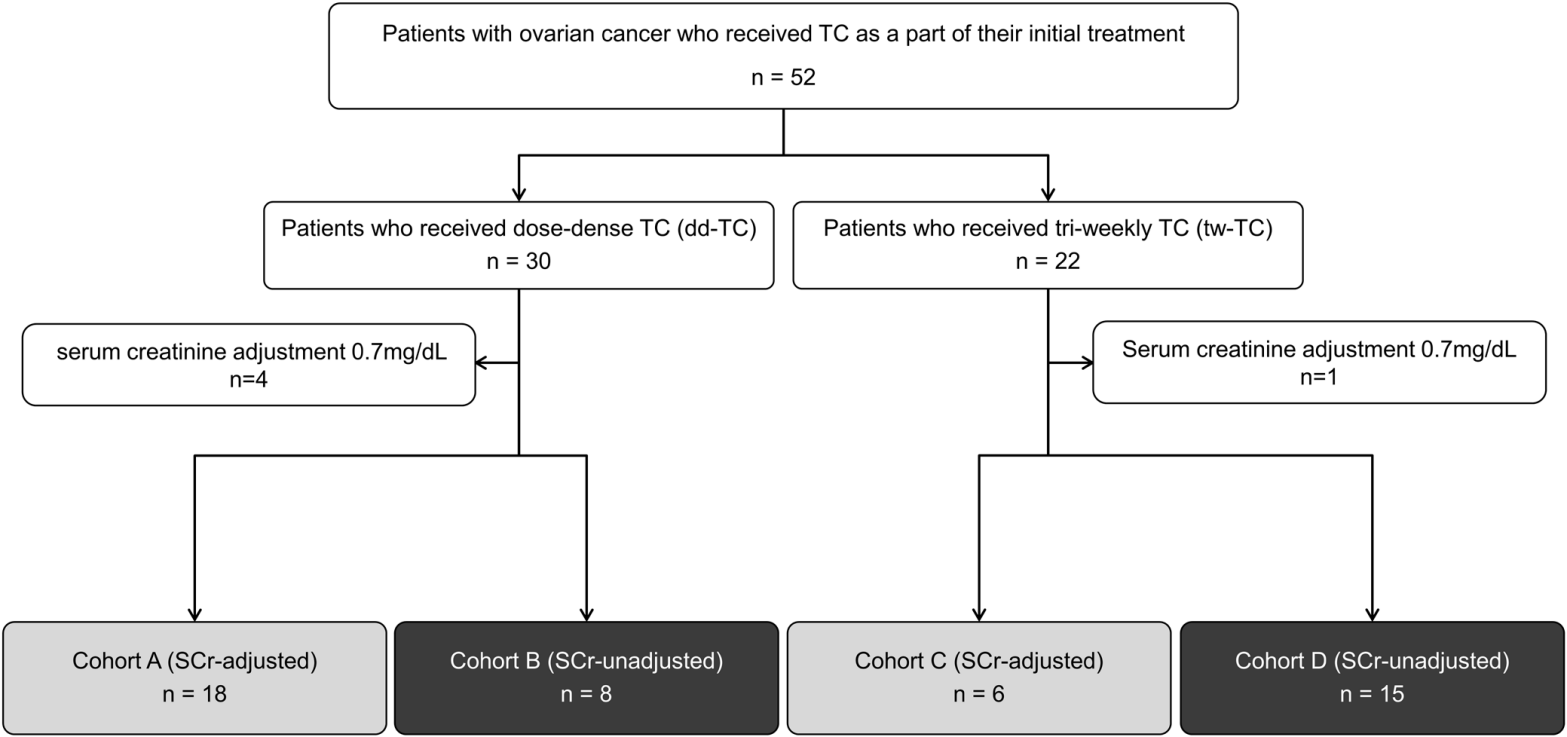
Classification of the cohorts. Patients who were eligible for the study were divided into four cohorts according to their treatment (dd‐TC or tw‐TC) and with or without SCr adjustment at the time of CBDCA dosing design.

Patients were categorized into four cohorts based on the choice of TC regimen, dd‐TC or tw‐TC, and presence or absence of SCr‐adjustment: Cohort A, dd‐TC with SCr‐adjustment; Cohort B, dd‐TC without SCr‐adjustment; Cohort C, tw‐TC with SCr‐adjustment; and Cohort D, tw‐TC without SCr‐adjustment (Figure 1). The DI and other DI‐related outcome measures defined below were compared between Cohorts A and B and Cohorts C and D. The timing of the first dose reduction, postponement, or skipping, the reasons for these, and adverse events were also compared between the cohorts.

### 2.3 Outcome Measures

DI:

PTX (mg/m^2^/week): actual total dose (mg)/body surface area (m^2^)/duration of treatment (weeks)

CBDCA (mg/h/L/week): target AUC (mg/mL × min) based on unadjusted* SCr/duration of treatment (weeks)

*In Cohorts A and C, the AUC was recalculated using the unadjusted SCr from the CBDCA dose actually administered.

Relative Dose Intensity (RDI) (%):

Actual DI/Planned DI**

**Planned DIs for dd‐TC and tw‐TC were calculated as follows.

dd‐TC: PTX (80 mg/m^2^) on Days 1, 8, and 15 and CBDCA (target AUC = 6 mg/mL × min) on Day 1, every 3 weeks; planned DIs of PTX and CBDCA, 80 mg/m^2^/week and target AUC 2 mg/mL × min/week, respectively.

tw‐TC: PTX (175 mg/m^2^) on Day 1 and CBDCA (target AUC = 6 mg/mL × min) on Day 1, every 3 weeks; planned DIs of PTX and CBDCA, 58.3 mg/m^2^/week and target AUC 2 mg/mL × min/week, respectively.

Average Relative Dose Intensity (ARDI) (%):

(RDI of CBDCA + RDI of PTX)/2.

#### 2.3.1 Total Treatment Period

The total treatment period was defined as cycle 1 Day 1 to cycle 6 Day 22. Deviation from the scheduled total treatment period of 126 days was indicated as a positive number if extended and as a negative number if shortened.

#### 2.3.2 Treatment Modification Rate

The treatment modification rate was defined as the proportion of patients who required dose reduction, postponement, or skipping of TC therapy to manage adverse events. In addition, the time to the initial treatment modification was also compared in an exploratory analysis.

### 2.4 Statistical Analysis

The *χ* ^2^ test was used for nominal variables and the Wilcoxon rank‐sum test for continuous variables, with and without SCr adjustment. The effect of SCr adjustment on the time to initial treatment modification was compared using the Kaplan–Meier method. *p* < 0.05 was considered statistically significant. Statistical data were analyzed using SAS Institute Inc. JMP: Statistical Software 11.2 (NC, US).

### 2.5 Ethics

This study was approved by the Ethics Review Board of the National Cancer Center following the “Ethical Guidelines for Medical and Health Research Involving Human Subjects” (approval number: 2022‐291). The data were anonymized and personal information was kept confidential. The IRB waived the requirement for obtaining written informed consent from the study subjects.

## 3 Results

### 3.1 Patient Background and Length of Treatment

A total of 47 patients were included in the study, 18 in Cohort A, 8 in Cohort B, 6 in Cohort C, and 15 in Cohort D (Table 1). The patients' baseline characteristics including age, height, and weight were similar between the cohorts, and all had PS ≤ 2. Patients who received dd‐TC, that is, Cohorts A and B, tended to have more advanced pStage than those who received tw‐TC. The SCr level used for the dose setting of CBDCA was generally similar among the cohorts (Table 1).

**TABLE 1:** Patient characteristics at baseline.

	dd‐TC cohorts		tw‐TC cohorts	
	Cohort A (*n* = 18)	Cohort B (*n* = 8)	Cohort C (*n* = 6)	Cohort D (*n* = 15)
Age (years), median (range)	61 (33–74)	62.5 (45–72)	60 (50–70)	56 (23–74)
Height (cm), median (range)	156. 3 (139. 3–166.4)	155.1 (152. 2–160.2)	153.6 (143.2–160.0)	155.7 (144.2–161.2)
Weight (kg), median (range)	48.8 (32.4–67.4)	43.8 (39.4–64.7)	52.9 (39.4–62.7)	51.0 (39.9–62.4)
ECOG PS, *n* (%)				
0	0 (0)	0 (0)	6 (100)	12 (80)
1	12 (67)	2 (25)	0 (0)	3 (20)
2	6 (33)	6 (75)	0 (0)	0 (0)
pStage, *n* (%)				
I	0 (0)	1 (13)	6 (100)	10 (67)
II	5 (28)	1 (13)	0 (0)	1 (7)
III	9 (50)	4 (50)	0 (0)	3 (20)
IV	4 (22)	2 (25)	0 (0)	1 (7)
SCr at dose design (mg/dL), median (range)	0.58 (0.49–0.74)	0.59 (0.53–0.72)	0.57 (0.44–0.76)	0.61 (0.49–0.95)
Unadjusted creatinine clearance (mL/min), median (range)	78.7 (44.5–127.3)	82.7 (61.4–94.8)	72.9 (59.2–123.0)	76.5 (56.6–124.8)

Abbreviations: dd‐TC, dose‐dense carboplatin + paclitaxel; ECOG PS, Eastern Cooperative Oncology Group Performance Status; pStage, pathological stage; SCr, serum creatinine; tw‐TC, tri‐weekly carboplatin + paclitaxel.

### 3.2 DI‐Related Outcome Measures

#### 3.2.1 DIs

In the cohorts of dd‐TC, there was no significant difference in the DIs of CBDCA between Cohorts A and B (median DI of CBDCA [target AUC/week], Cohort A vs. B, 1.43 vs. 1.40, *p* = 0.76) (Table 2). Conversely, the DI of PTX was significantly higher in Cohort A than in Cohort B (median DI of PTX [mg/m^2^/week], Cohort A vs. B, 71.4 vs. 49.8, *p* = 0.002) (Table 2). In contrast, in the cohorts of tw‐TC, there was no significant difference in the DI of CBDCA or PTX between Cohorts C and D (median DI of CBDCA [target AUC/week], Cohort C vs. D, 1.48 vs. 1.65, *p* = 0.23; median DI of PTX [mg/m^2^/week], Cohort C vs. D, 53.4 vs. 56.9, *p* = 0.37) (Table 2).

**TABLE 2:** Treatment intensity for each cohort.

	dd‐TC cohorts		*p*	tw‐TC cohorts		*p*
	Cohort A (*n* = 18)	Cohort B (*n* = 8)		Cohort C (*n* = 6)	Cohort D (*n* = 15)	
CBDCA DI, AUC/week, median (range)	1.43 (0.89–1.79)	1.40 (0.99–1.80)	0.760	1.48 (1.38–1.67)	1.65 (1.14–1.99)	0.230
PTX DI, mg/m^2^/week, median (range)	71.4 (32.2–84.9)	49.8 (15.0–71.7)	0.002	53.4 (57.0–51.7)	56.9 (41.0–58.5)	0.370
CBDCA RDI, %, median (range)	89.9 (60.0–105.9)	78.3 (53.9–89.9)	0.005	94.8 (88.7–100.4)	98.8 (62.2–100.0)	0.560
PTX RDI, median % (range)	89.2 (40.2–106.1)	62.2 (18.8–89.7)	0.002	91.5 (88.7–97.8)	97.5 (70.3–100.2)	0.350
CBDCA RDI ≥ 85%, *n* (%)	14 (78)	1 (13)	0.002	6 (100)	13 (87)	0.340
PTX RDI ≥ 85%, *n* (%)	14 (78)	1 (13)	0.002	6 (100)	13 (87)	0.350
ARDI, median % (range)	83.9 (45.7–100.1)	70.2 (50.2–89.8)	0.002	93.1 (88.7–99.1)	98.3 (66.3–100.0)	0.700
ARDI ≥ 85%, *n* (%)	14 (78)	1 (13)	0.002	6 (100)	13 (87)	0.340

Abbreviations: ARDI, average relative dose intensity; CBDCA, carboplatin; dd‐TC, dose‐dense carboplatin + paclitaxel; DI, dose intensity; PTX, paclitaxel; RDI, relative dose intensity; tw‐TC, tri‐weekly carboplatin + paclitaxel.

#### 3.2.2 RDI and ARDI

In the cohorts of dd‐TC, the RDIs (%) for both drugs were higher in Cohort A than in Cohort B (median RDI of CBDCA [%], Cohort A vs. B, 89.9 vs. 78.3, *p* = 0.005; median RDI of PTX [%], Cohort A vs. B, 89.2 vs. 62.2, *p* = 0.002) (Table 2). Consistently, the proportions of patients with RDI ≥ 85% (%) were also higher in Cohort A than in Cohort B for both drugs (proportion of patients with RDI ≥ 85% [%] for CBDCA, Cohort A vs. B, 78 vs. 13, *p* = 0.002; proportion of patients with RDI ≥ 85% [%] for PTX, Cohort A vs. B, 78 vs. 13, *p* = 0.002), and this trend was also observed for ARDI (Table 2). Conversely, in the cohorts of tw‐TC, there was no difference in RDIs, the proportions of patients with RDI ≥ 85% (%) for either drug, or ARDI ≥ 85% (%) (Table 2).

### 3.3 Compliance and Safety Measures

**FIGURE 2:**
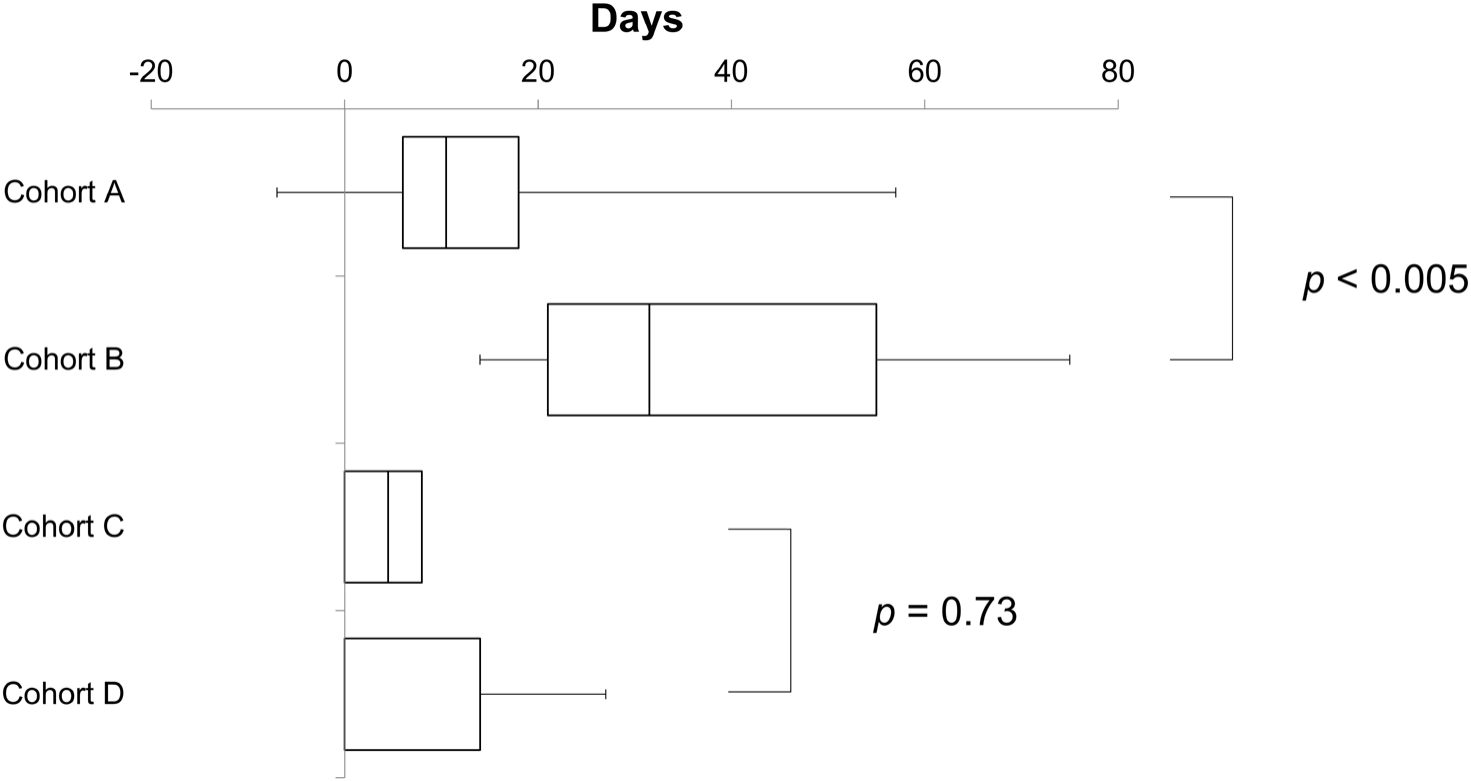
Total treatment period. The planned total treatment period of 126 days was set to 0 days. Deviations were expressed as a positive number in the case of prolongation and a negative number in the case of shortening.

The total treatment period for each cohort is shown in Figure 2. In the cohorts of dd‐TC, the total treatment period was significantly longer in Cohort A than in Cohort B (median extended days, Cohorts A vs. B, +31.5 vs. +10.6 days, *p* = 0.004). However, in the cohorts of tw‐TC, there was no significant difference in the total treatment period between the two cohorts (median extended days, Cohorts C vs. D, +4.5 vs. 0 days, *p* = 0.73).

The treatment modification rates for Cohorts A and B were 89% and 100%, respectively. Notably, all patients in Cohort B required treatment modification by the third cycle (Figure S1). The most common reason for the first dose reduction was hematologic toxicity in dd‐TC, with neutropenia being the most common. In Cohort A, of the eight cases, two cases had neutropenia, one case had thrombocytopenia, and one case had anemia that led to a dose reduction (Table S1). In Cohort B, of five patients with a dose reduction, three had neutropenia and two had elevated AST and ALT (Table S1). However, there were no cases of dose reduction due to hematologic toxicity in the tw‐TC cohorts (Table S1). In the cohorts of tw‐TC, Cohorts C and D had treatment modification rates of 0% and 33%, respectively, indicating fewer treatment modifications in patients with SCr adjustment (Figure S1).

### 3.4 Adverse Events

In the cohort of dd‐TC, Cohorts A and B, the incidence of grade ≥ 3 neutropenia was significantly higher in Cohort B than in Cohort A (Cohorts A vs. B, 17 vs. 100%, *p* < 0.005) (Table 3) even though the incidence of neutropenia in any grade was similar between the two groups (Cohort A vs. B, 89 vs. 100%, *p* = 0.326). No patients in this study received G‐CSF. Thrombocytopenia tended to occur more frequently in Cohort B when including any grade, but grade ≥ 3 thrombocytopenia was infrequent in either cohort with no statistically significant difference (Cohort A vs. B, any grade thrombocytopenia, 17 vs. 75%, *p* = 0.004; grade ≥ 3 thrombocytopenia, 6 vs. 0%, *p* = 0.500) (Table 3). In the cohort of tw‐TC, Cohorts C and D, there was a trend toward a higher incidence of grade ≥ 3 neutropenia in Cohort D, although it did not reach a statistically significant difference (Cohort C vs. D, any grade neutropenia, 67 vs. 73%, *p* = 0.760; grade ≥ 3 neutropenia, 0 vs. 27%, *p* = 0.160). In Cohort C, only one patient had grade 2 thrombocytopenia and no grade ≥ 3 thrombocytopenia was observed.

**TABLE 3:** Worst grades for hematologic toxicities over the entire treatment period.

	dd‐TC cohorts		*p*	tw‐TC cohorts		*p*
	Cohort A (*n* = 18)	Cohort B (*n* = 8)		Cohort C (*n* = 6)	Cohort D (*n* = 15)	
Neutropenia all grades, *n* (%)	16 (89)	8 (100)	0.326	4 (67)	11 (73)	0.760
Neutropenia grade ≥ 3, *n* (%)	5 (17)	8 (100)	< 0.005	0 (0)	4 (27)	0.160
Thrombocytopenia all grades, *n* (%)	3 (17)	6 (75)	0.004	1 (17)	0 (0)	—
Thrombocytopenia grade ≥ 3, *n* (%)	1 (6)	0 (0)	0.500	0 (0)	0 (0)	—

Abbreviations: dd‐TC, dose‐dense carboplatin + paclitaxel; tw‐TC, tri‐weekly carboplatin + paclitaxel.

## 4 Discussion

To the best of our knowledge, this study represents the first investigation of the impact of the adjustment of SCr by adding 0.2 mg/mL to that measured by enzyme methods on the perioperative treatment of ovarian cancer. We found that the SCr adjustment in dd‐TC cohorts was associated with a significantly higher DI of PTX, RDI of PTX and CBDCA, and ARDI compared with no adjustment with equivalent DIs of CBDCA. Conversely, there was no significant difference in the DI, RDI, or ARDI between tw‐TC with and without adjustment.

Adjusting the SCr by 0.2 mg/mL results in a lower estimate of renal function, and therefore a lower dose setting of CBDCA using the Calvert formula. The higher DI of PTX in the dd‐TC cohorts despite the lower CBDCA dose setting in the adjustment cohort was likely attributable to higher compliance with the dosing schedule. Indeed, Cohort A had a higher RDI and ARDI than Cohort B, suggesting that the overall treatment tended to be on schedule. Consistently, the total treatment period was significantly shorter in Cohort A than in Cohort B (Figure 2). Although the treatment modification rates for Cohorts A and B were both high, 89% and 100%, respectively (Figure S1), Cohort A had less frequent dose reduction, postponement, or skipping during the first three cycles compared with Cohort B (Figure S1), resulting in a higher total DI. Also of note, five of eight patients in Cohort B who required a dose reduction during the first three cycles had a reduced dose of CBDCA from the target AUC of 6 to 5. After recalculating the initial dose of CBDCA in Cohort A using the unadjusted SCr, we found it was equivalent to the median target AUC of 4.82. This suggested that even if patients were started on a higher dose of CBDCA based on the unadjusted SCr, it would eventually need to be reduced to the same level as that after the adjustment in many patients. In a study of patients with lung cancer, the same adjustment of +0.2 mg/dL to the SCr by enzymatic methods in the dose setting of CBDCA was consistently shown to better maintain the RDI of CBDCA [16] and to reduce toxicity without compromising treatment efficacy [17].

In contrast to the cohorts of dd‐TC, no significant differences in the DI, RDI, or ARDI were observed in the cohorts of tw‐TC, Cohorts C and D. This might be related to the fact that the scheduled DI of PTX was lower in tw‐TC than in dd‐TC, 58 vs. 80 mg/m^2^/week. In addition, in the dd‐TC where blood tests were typically performed on Days 8 and 15 before the administration of PTX, tw‐TC only performs a blood test on Day 1 of each cycle. Therefore, neutropenia and thrombocytopenia are less likely to be detected compared with those in the dd‐TC cohort, and fewer treatment modifications may have been performed regardless of the SCr adjustment.

The main reason for a treatment dose reduction, postponement, or skip in this study was myelosuppression, especially neutropenia. Notably, the incidence of grade ≥ 3 neutropenia was significantly higher in Cohort B compared with Cohort A (100% vs. 20%) (Table 3). A large phase III trial examining the efficacy of dd‐TC reported grade ≥ 3 neutropenia in more than 90% of patients assigned to the dd‐TC arm, which was comparable with the incidence of grade ≥ 3 neutropenia in Cohort B in our study [7]. Therefore, it appears that SCr adjustment has a marked impact on reducing the incidence of grade ≥ 3 neutropenia. We hypothesized that thrombocytopenia might be more common in Cohort B than A and in Cohort D than C, because it is a dose‐limiting toxic effect of CBDCA. But only one patient actually experienced grade ≥ 3 thrombocytopenia in all the cohorts and the patient was treated with dd‐TC with SCr adjustment. This trend toward a greater impact on neutropenia compared with thrombocytopenia was observed in a previous study of lung cancer patients that examined the impact of SCr adjustment on the CBDCA dosing design: the incidence of grade ≥ 3 neutropenia was significantly lower in the adjusted group (with/without adjustment; 13/30%, *p* = 0.03), but thrombocytopenia was similar between the two groups (6/12%, *p* = 0.28) [18]. This phenomenon may be related to the “platelet sparing effect” of PTX because the co‐administration of PTX inhibited CBDCA‐induced thrombocytopenia [19, 20].

This study had several limitations. First, it did not examine the parameters directly related to treatment efficacy, such as overall survival or recurrence‐free survival. This study included only post‐PDS patients, and tumor responses could not be assessed because of the absence of evaluable lesions. In addition, because we began the SCr adjustment in 2020 based on a team consensus, more patients in Cohorts A and C started their treatment after the extensive use of PARP inhibitors as maintenance therapy became available, which made it more difficult to evaluate the impact of SCr adjustment on the efficacy measured by time‐dependent endpoints, including progression‐free survival. RDI < 85% and ARDI < 85% were reported to be associated with poor overall survival in previous studies [8, 21], and therefore we used these metrics as surrogates for time‐dependent endpoints. However, whether higher RDI and ARDI by SCr adjustment are associated with a better prognosis remains to be verified in larger studies.

Second, this study was based on clinical practice in the real world, and this does not fully follow management strategies used in clinical trial settings. As an example, dd‐TC was reported to be a feasible regimen in clinical trials, including one implemented in Japan whose protocol stipulated that the SCr should be measured with enzymatic methods but did not specifically recommend the adjustment of SCr. Although the actual DIs of CBDCA and PTX were not reported [7], they may have been maintained by the administration of granulocyte colony stimulating factor (G‐CSF) during neutropenia, which the study protocol left to the physician's decision. Indeed, it was reported that G‐CSF administration was necessary in approximately 60% of cases [7]. However, it is not practical to force patients with neutropenia to attend the hospital every day for the administration of G‐CSF under circumstances where pegylated G‐CSF is not feasible because of the administration of PTX on Days 8 and 15. Third, this was a retrospective observational study with a limited number of cases, which could have limited the statistical power. In addition, there were no standardized criteria for dose modification or skipping of chemotherapy because of toxicity, which was clinically determined by the treating physician. However, we think that the findings of this study reflect the reality of clinical practice, where treatments are tailored to the specific needs of individual patients. Fourth, the dd‐TC regimen is not universally adopted because phase III studies conducted after the Japanese study failed to demonstrate the superiority of dd‐TC over tw‐TC [22]. However, dd‐TC is still recognized as a standard treatment in the Japanese guidelines and is commonly used as first‐line therapy in Japan [2]. Despite these limitations, this study provides valuable data suggesting that SCr adjustment in clinical practice may help maintain DI by reducing adverse events and treatment modifications.

In conclusion, the adjustment of SCr by adding 0.2 mg/dL appears to contribute to implementing dd‐TC on schedule and consequently to maintain the DI of PTX, RDI of CBDCA and PTX, and ARDI at high levels. Prospective studies or larger cohort studies using these efficacy measures are warranted to validate the true value of the adjustment.

## Author Contributions

**Shu Kato:** conceptualization (equal), formal analysis (equal), investigation (equal), methodology (equal), project administration (equal), writing – original draft (equal), writing – review and editing (equal). **Kaede Baba:** conceptualization (equal), methodology (equal), project administration (equal), writing – original draft (equal), writing – review and editing (equal). **Kanako Mamishin:** conceptualization (equal), writing – review and editing (equal). **Mao Uematsu:** writing – review and editing (equal). **Mai Shimura:** conceptualization (equal), writing – review and editing (equal). **Akira Hirota:** writing – review and editing (equal). **Misao Fukuda:** conceptualization (equal), writing – review and editing (equal). **Nobuyuki Takahashi:** conceptualization (equal), writing – review and editing (equal). **Takehiro Nakao:** writing – review and editing (equal). **Hiromichi Nakajima:** conceptualization (equal), writing – review and editing (equal). **Chikako Funasaka:** conceptualization (equal), writing – review and editing (equal). **Chihiro Kondoh:** conceptualization (equal), writing – review and editing (equal). **Kenichi Harano:** conceptualization (equal), writing – review and editing (equal). **Yoichi Naito:** writing – review and editing (equal). **Nobuaki Matsubara:** writing – review and editing (equal). **Ako Hosono:** writing – review and editing (equal). **Toshikatsu Kawasaki:** supervision (equal), writing – review and editing (equal). **Toru Mukohara:** conceptualization (equal), methodology (equal), writing – original draft (equal), writing – review and editing (equal).

## Ethics Statement

The research protocol for this study was approved by the Ethics Review Board of the National Cancer Center following the “Ethical Guidelines for Medical and Health Research Involving Human Subjects” (approval number: 2022‐291).

## Consent

The IRB waived the requirement for obtaining written informed consent from the study subjects.

## Conflicts of Interest

The authors declare no conflicts of interest.

## Supporting information


**Figure S1.** Timing of the initial treatment modification in each cohort.

**Table S1.** Reasons for the first dose reduction in each cohort.

## Data Availability

The data that support the findings of this study are available from the corresponding author upon reasonable request.
