# Supplementary figures and images for: The Impact of Carboplatin Dosing Design Using Adjusted Serum Creatinine on Carboplatin Plus Paclitaxel Therapy for Ovarian Cancer
Source: Cancer Med. 2025 Mar 27;14(7):e70804. doi: 10.1002/cam4.70804 (PMC11947989; doi:10.1002/cam4.70804)

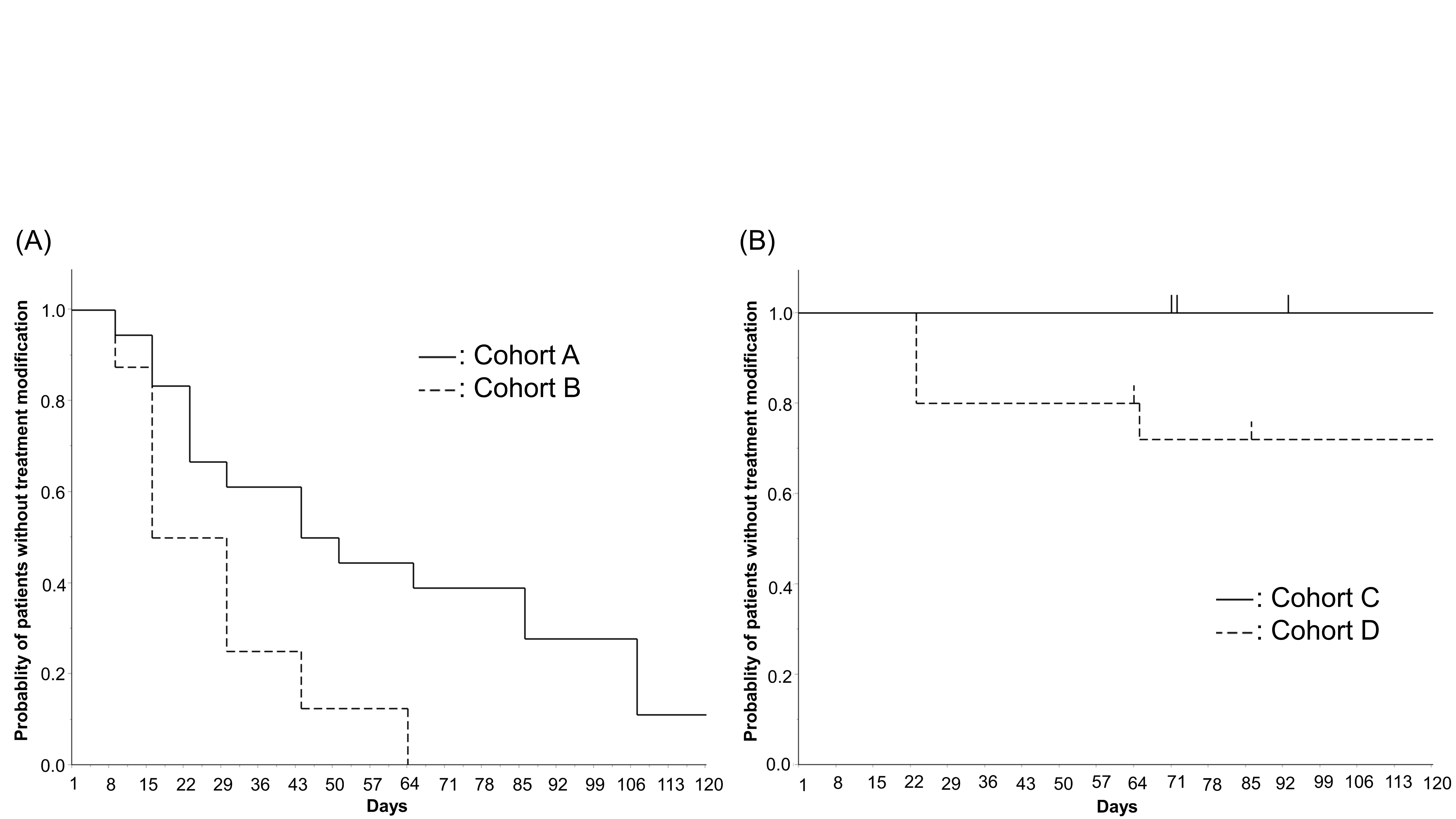

Supplement: Supplementary file 1 — Figure S1. Timing of the initial treatment modification in each cohort. [file CAM4-14-e70804-s001.tif]
