# Supplementary material for: The Impact of Carboplatin Dosing Design Using Adjusted Serum Creatinine on Carboplatin Plus Paclitaxel Therapy for Ovarian Cancer
Source: Cancer Med. 2025 Mar 27;14(7):e70804. doi: 10.1002/cam4.70804 (PMC11947989; doi:10.1002/cam4.70804)
Supplement: Supplementary file 2 — Table S1. Reasons for the first dose reduction in each cohort. [file CAM4-14-e70804-s002.docx]

| **Supplemental Table 1.** Reasons for the first dose reduction in each cohort | | | | |
| --- | --- | --- | --- | --- |
|  | **dd-TC cohorts** | | **tw-TC cohorts** | |
|  | Cohort A (n = 18) | Cohort B  (n = 8) | Cohort C  (n = 6) | Cohort D  (n =15) |
| Neutropenia | 3 | 3 | 0 | 0 |
| Thrombocytopenia | 1 | 0 | 0 | 0 |
| Anemia | 1 | 0 | 0 | 0 |
| Elevated AST or ALT | 0 | 2 | 0 | 0 |
| Intestinal obstruction | 0 | 0 | 0 | 1 |
| Fatigue | 1 | 0 | 0 | 0 |
| Elevated serum creatinine | 0 | 0 | 0 | 1 |
| Unidentified | 2 | 0 | 0 | 0 |
| Total | 8 | 5 | 0 | 2 |
| dd-TC, dose-dense carboplatin + paclitaxel; tw-TC, tri-weekly carboplatin + paclitaxel ALT, alanine aminotransferase; AST, aspartate aminotransferase | | | | |
